# Dual Biomembrane Force Probe enables single-cell mechanical analysis of signal crosstalk between multiple molecular species

**DOI:** 10.1038/s41598-017-13793-3

**Published:** 2017-10-27

**Authors:** Lining Ju, Yunfeng Chen, Kaitao Li, Zhou Yuan, Baoyu Liu, Shaun P. Jackson, Cheng Zhu

**Affiliations:** 1 Coulter Department of Biomedical Engineering, Georgia Institute of Technology, Atlanta, 30332 GA United States; 2 Petit Institute for Bioengineering and Biosciences, Georgia Institute of Technology, Atlanta, 30332 GA USA; 3 Woodruff School of Mechanical Engineering, Georgia Institute of Technology, Atlanta, 30332 GA USA; 4 Heart Research Institute, Newtown, 2050 NSW Australia; 5 Charles Perkins Centre, The University of Sydney, Camperdown, 2006 NSW Australia; 6 Department of Molecular Medicine, MERU-Roon Research Center on Vascular Biology, The Scripps Research Institute, La Jolla, 92037 CA USA

## Abstract

Conventional approaches for studying receptor-mediated cell signaling, such as the western blot and flow cytometry, are limited in three aspects: 1) The perturbing preparation procedures often alter the molecules from their native state on the cell; 2) Long processing time before the final readout makes it difficult to capture transient signaling events (<1 min); 3) The experimental environments are force-free, therefore unable to visualize mechanical signals in real time. In contrast to these methods in biochemistry and cell biology that are usually population-averaged and non-real-time, here we introduce a novel single-cell based nanotool termed dual biomembrane force probe (dBFP). The dBFP provides precise controls and quantitative readouts in both mechanical and chemical terms, which is particularly suited for juxtacrine signaling and mechanosensing studies. Specifically, the dBFP allows us to analyze dual receptor crosstalk by quantifying the spatiotemporal requirements and functional consequences of the up- and down-stream signaling events. In this work, the utility and power of the dBFP has been demonstrated in four important dual receptor systems that play key roles in immunological synapse formation, shear-dependent thrombus formation, and agonist-driven blood clotting.

## Introduction

Over the past decade, single-molecule biomechanical analyses on single cells have enabled studies of the inner workings of adhesion and signaling receptors one at a time^1^. In physiological conditions, however, multiple receptor species are present and often work cooperatively rather than independently. Understanding how multiple receptor species crosstalk to each other is essential for determining the mechanisms underlying a wide range of biological processes related to human health and diseases. One of the model receptor systems that exhibit such signaling crosstalk with other receptors is the integrin family. For example, integrin α_L_β_2_, or lymphocyte function-associated antigen-1 (LFA-1), crosstalks with chemokine receptors and antigen receptors via an “inside-out” signaling process^2^, which is essential for lymphocyte trafficking and immunological synapse formation^3^. The interaction between LFA-1 on a lymphocyte and its ligand, intercellular adhesion molecule-1 (ICAM-1) on an adjacent cell, can be upregulated by signals induced by binding of chemokines and antigens to their respective receptors on the same lymphocyte, manifesting enhanced cell adhesion^4,5^. As another example, upon a rapid shear force increase caused by blood flow perturbations, the function of integrin α_IIb_β_3_, or glycoprotein (GP) IIb-IIIa, on a platelet surface is rapidly upregulated by signals induced by ligand engagement with the mechanoreceptor GPIb^6,7^, which promotes platelet adhesion at the site of vascular injury as part of the hemostatic and thrombotic processes^8^. The synergistic binding of GPIb and GPIIb-IIIa enables efficient platelet recruitment under hemodynamic shear, thereby allowing further platelet activation by physiological agonists (i.e. ADP) to stabilize the thrombus^8^.

To study the molecular details regarding how ligand binding and signaling of the aforementioned (TCR/LFA-1) and (GPIb/GPIIb-IIIa) dual receptor systems are orchestrated in time and space in their corresponding physiological processes, it requires methods and techniques capable of doing so. Two types of methods have been developed for manipulating, visualizing, and quantitatively analyzing receptor–ligand interactions on single cell in real-time^9,10^. One type is mechanically based, using pico-force techniques, such as the commercially available atomic force microscope and optical traps^11^ as well as the user-made micropipette-based biomembrane force probe (BFP)^1,12,13^. The other type is fluorescently based, using sensitive imaging systems, such as that used on hybrid junctions between live cells and supported lipid bilayer^14,15^.

Most studies using the mechanically based approaches are limited to biological systems involving single receptor–ligand species. Only in a few cases have systems involving dual species of receptors, ligands, or both been studied. Interactions of two ligands (IgG1 and IgG2) to a common receptor (Fcγ receptor III B, FcγRIIIB)^16^ or of two receptors (FcγRIIA and FcγRIIIB) to a common ligand (total IgG)^17^ have been found to be concurrent and independent when the bond numbers are low enough for competition to be neglected, resulting in a total bond number that is equal to the sum of two bond species. By comparison, binding of a divalent ligand, pMHC or Thy-1, to the corresponding dual receptors, T cell receptor (TCR) and coreceptor CD8^18–20^ or integrin α_5_β_1_ and Sydecan-4^21^, is cooperative and synergistic, resulting in an additional trimolecular bond species of greater number and longer duration than the sum of two bimolecular bond species separately formed between the ligand and the two receptors. Interestingly, the formation of the (TCR/CD8)–pMHC trimolecular bond exhibits a time delay and requires intracellular signaling via Src family kinases^18–20^ whereas that of the (α_5_β_1_/Sydecan-4)–Thy-1 bond is elicited by pulling forces^21^. The mechanically based methods have also been combined with fluorescence imaging to enable concurrent observations of receptor–ligand binding and intracellular signaling so induced, to allow for correlative analysis over time at the single-cell level^22–24^. A major limitation of the existing mechanically based methods is the inability to control the separate engagement of multiple receptor–ligand species.

Fluorescently based approaches have been widely used to visualize the (co)localization and movement of multiple molecular species by multi-color imaging. They have also been employed to study dual receptor cooperativity by co-presenting two ligands (i.e. pMHC/ICAM-1) to two receptors (i.e. TCR/LFA-1) on the same cell^25^. In addition, fluorescently based approaches have been combined with nanoprint lithography to present multiple ligands in localized and separated patterns to control the engagement of cell surface receptors in space^26,27^. Major limitations of the existing fluorescently based methods include the inability to exert controlled forces on receptor–ligand bonds of interests as well as the difficulty and low throughput of the single-bond kinetic analysis. Therefore, studies of binding, and signaling crosstalk of multiple receptor–ligand species are limited by methods.

To address these issues, here we describe the development of a mechanically based method – dual biomembrane force probe (dBFP), which allows us to examine the signal reception, initiation and transduction from one receptor to another on a single cell step by step in space and time. The utility and power of dBFP were demonstrated in four important dual receptor systems: (TCR/LFA-1), (GPIb/GPIIb-IIIa), (GPIb/CD62p), and (P_2_Y/CD62p). The first system plays a key role in immunological synapse formation^3^; the next two systems play key roles in shear-dependent thrombus formation^8,28^; and the last system plays a key role in agonist-driven blood clotting^8,29^.

## Results

**Figure 1:**
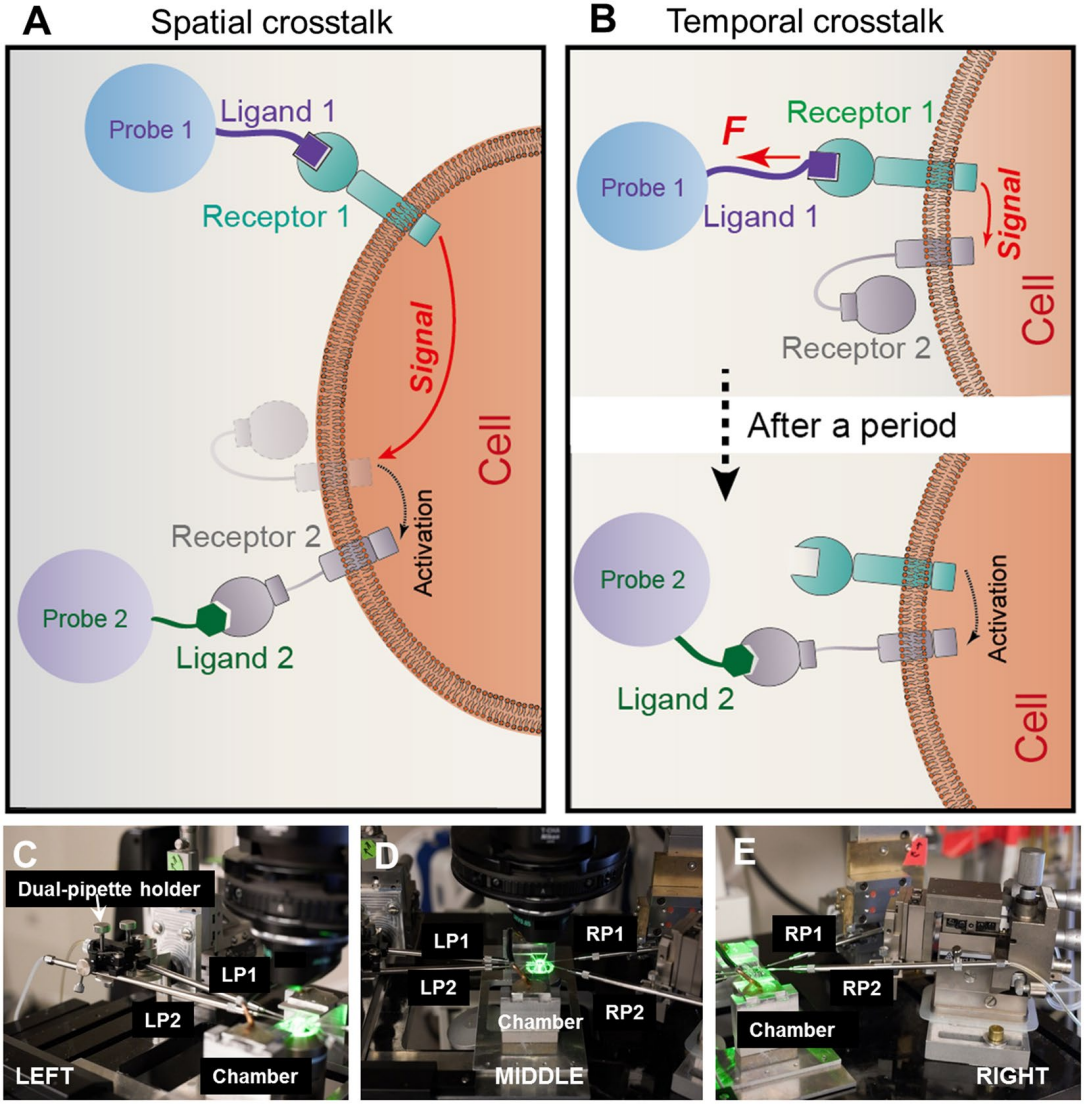
Concept and instrumentation of the dual biomembrane force probe (dBFP). (**A**) Schematic of a spatial crosstalk dual receptor system. Ligands 1 and 2 are presented by the respective Probes 1 and 2 to the cell at spatially separate locations. The signal initiated upon binding of Receptor 1 at one location travels a distance to activate Receptor 2 at another location. (**B**) Schematic of a temporal crosstalk dual receptor system. Ligands 1 and 2 are presented by the respective Probes 1 and 2 to the cell at temporally separate times. The signal initiated upon the binding of Receptor 1 upregulates Receptor 2, which remains upregulated after a period. (**C–E**) Images of the hardware system, consisting of two micropipettes (LP1, LP2) on the left side (**C** and **D**), the reaction chamber (**C**–**E**) and the other two micropipettes (RP1, RP2) on the right side (**D** and **E**).

One type of dual receptor crosstalk manifests as the expression or binding affinity of one receptor being upregulated upon signals triggered by another receptor. The two receptors may be separated in space (Fig. 1A), and the signaling process may take time (Fig. 1B). To investigate the spatiotemporal requirements of this type of dual receptor crosstalk, weconfigured the dBFP system with four micropipettes (Fig. 1C–E and Supplementary Fig. S1), two on the left (LP1 and LP2, Fig. 1C) and the other two on the right (RP1 and RP2, Fig. 1E), all inserting into the reaction chamber in the middle (Fig. 1D). Cells expressing the receptors of interest are to be tested in the reaction chamber against surface-bound or soluble ligands by well-controlled mechanical manipulations. Applications of the dBFP will be demonstrated by four examples as follows.

### Analyzing spatial crosstalk between TCR and LFA-1 on a T cell

**Figure 2:**
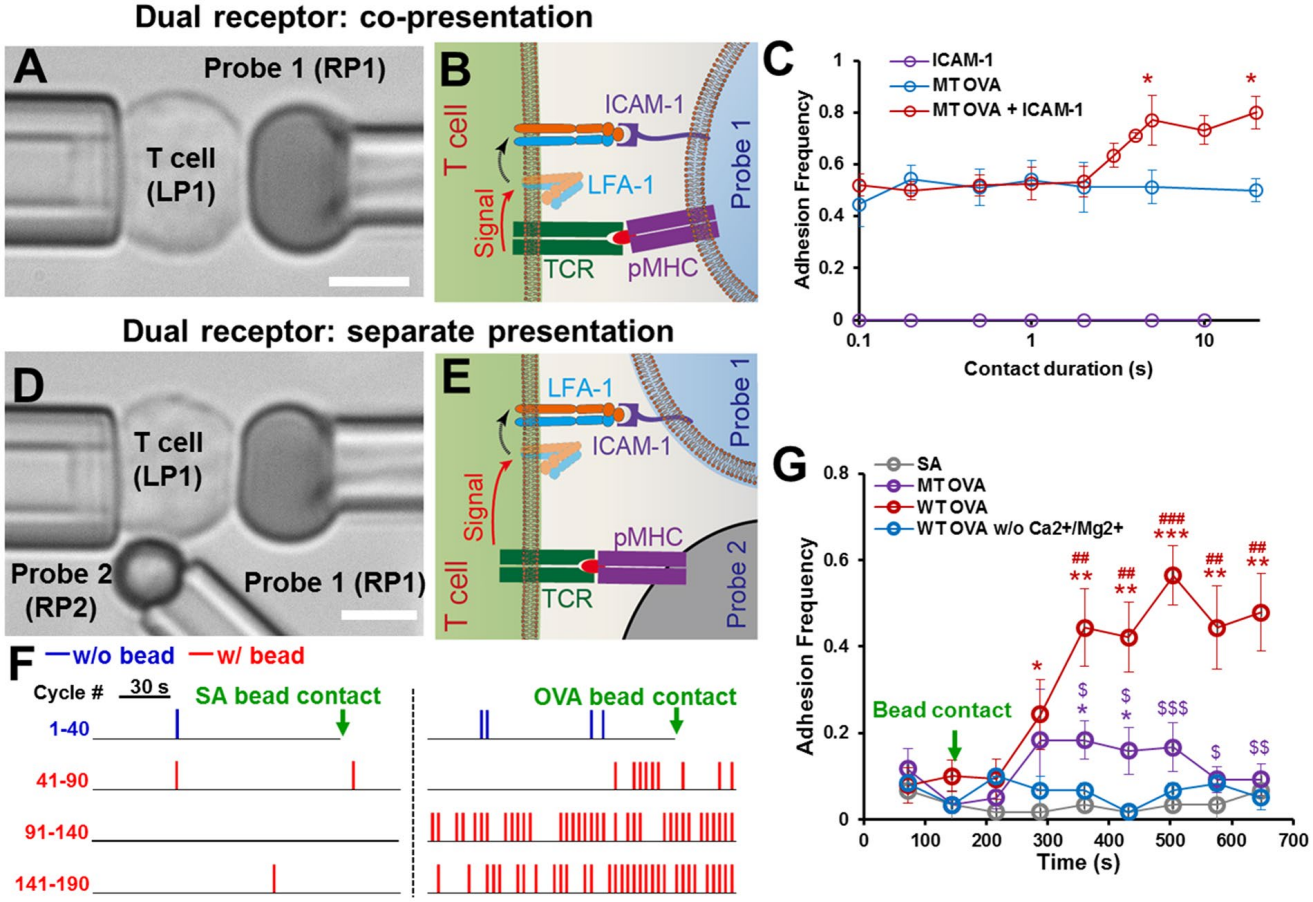
Analyzing spatial crosstalk between TCR and LFA-1. (**A–C**) Dual ligand co-presentation setup and results. A T cell aspirated by LP1 was repetitively touched by a functionalized RBC (Probe 1) aspirated by RP1 (**A**). Probe 1 was coated with a mixture of pMHC and ICAM-1 to engage TCR and LFA-1 simultaneously (**B**). Adhesion frequency vs. contact duration curves of naïve OT1 CD8^+^ T cells tested against Probe 1 coated with ICAM-1 alone (purple), mutant OVA (OVA:H2-K^b^α3A2) alone (blue), or a mixture of mutant OVA and ICAM-1 (C, red, n ≥ 3 cells for each point). (**D–G**) Dual ligand separate presentation setup and results. The setup is the same as in A, except the addition of a bead (Probe 2) aspirated by RP2 to make constant contact with the T cell at a distal site from the Probe 1 contact site (**D**). Binding to the ICAM-1-bearing Probe 1 reports the LFA-1 activity upregulated by TCR engagement with pMHC coated on Probe 2. (**E**). Representative spike trains of adhesion events between a T cell and an ICAM-1-bearing Probe 1 in 190 cycles. After the first 40 cycles of rare adhesions (blue spikes), Probe 2 coated with SA (left) or wild-type (WT) OVA (OVA:H2-K^b^) (right) was moved to constantly contact with the T cell (green arrow), and Probe 1 continued to touch the T cell repeatedly for another 150 cycles of similarly rare (left) or much more frequent (right) adhesions (red spikes) (**F**). Time course of averaged adhesion frequency between T cell and ICAM-1-bearing Probe 1 calculated by binning the raw adhesion sequences with a 20-touch (72 s) window (**G**). The number of cells tested were: SA = 3 (gray), mutant OVA = 6 (purple), WT OVA = 7 (red), and WT OVA without Ca^2+^/Mg^2+^  = 3 (blue). Symbols *, $ and # indicate different groups of comparisons: WT OVA/MT OVA vs. SA, WT OVA vs. mutant OVA, and WT OVA vs. WT OVA without Ca^2+^/Mg^2+^, respectively. Single, double and triple symbols denote *p* < 0.05, 0.01 and 0.001, respectively, by Student t-test. Scale bars are 5 μm in all micrographs.

LFA-1 dependent lymphocyte adhesion strengthening under antigen or chemokine receptor stimulation has been well demonstrated in population assays, i.e., static cell adhesion assay or flow cytometric analysis with antibodies recognizing activated LFA-1^4,5^. To provide a contrast, we first analyzed the dual receptor crosstalk between TCR and LFA-1 by the published adhesion frequency assay using the existing single-receptor force probe technique^30,31^. A naïve OT1 CD8^+^ T cell aspirated by LP1 was brought into repeated contact with a RBC (serving as a force probe) coated with a mixture of pMHC and ICAM-1 held by piezo-controlled RP1 (Fig. 2A). To avoid the complication of CD8 binding, the OT1 TCR specific peptide OVA_257–264_ was presented by the MHC domain swapping mutant, H2-K^b^α3A2, that replaced the α3 domain of mouse H2-K^b^ with that of human HLA-A2^32^. Adhesion frequency (*P*_*a*_) was calculated over 50 cycles of contact for each contact duration (*t*_*c*_) precisely set by programed piezo movement. The *P*_*a*_ curve measured over a range of *t*_*c*_ exhibits a two-stage pattern (Fig. 2C, red), similar to those observed in the studies of TCR/CD8 crosstalk^18–20^. Like the previous studies, this two-stage curve cannot be obtained by superposition of two single-stage curves each measured using the same batch of T cells contacted by RBCs coated with only one ligand species (Fig. 2C, blue and purple). The reason is that ICAM-1 binding was negligible when the affinity of LFA-1 was low and its affinity upregulation by signals received from TCR (Fig. 2B) required time. This explains the undetectable purple binding curve (ICAM-1 alone), the initial overlap between the red curve (before LFA-1 upregulation) and blue curve (pMHC alone), and the higher red curve (after LFA-1 upregulation) than blue curve after a time delay (Fig. 2C). Thus, the *P*_*a*_ curve for mixture coating at the two ends reflects the concurrent and independent binding of two receptor–ligand species, TCR–pMHC and LFA-1–ICAM-1, with the LFA-1 having a low affinity at *t*_c_ < 2 s and a higher affinity at *t*_c_ > 5 s. The middle portion (2 s < *t*_c_ < 5 s) contains temporal information about the interplay between the TCR outside-in signaling and LFA-1 inside-out signaling processes occurring at the proximity of the two receptors underneath the T cell-RBC interface.

We wished to determine whether the TCR-induced LFA-1 activation is a local autonomy for the pMHC-triggered TCRs only or a globally orchestrated process across the whole cell, a question difficult to address using the single probe system but easy to study using our new dBFP system. To do so we presented the T cell at physically separate sites with probes coated with one ligand species at a place (Fig. 2D,E). Binding between the OT1 naïve CD8^+^ T cell aspirated by LP1 and ICAM-1-bearing RBC (Probe 1) driven by RP1 was continuously monitored to read out changes in LFA-1 binding affinity in real time for >10 min (~200 cycles). At the same time, a pMHC-bearing bead (Probe 2) aspirated by RP2 was making continuous contact at a site on the T cell distal from the LFA-1–ICAM-1 reaction zone to induce TCR signaling (Fig. 2D,E). As illustrated by the spike train raw data for representative adhesion sequences (Fig. 2F), contacts by Probe 2 coated with streptavidin (SA) alone did not alter the low frequency binding of T cell to the ICAM-1 bearing Probe 1. In sharp contrast, making contact with OVA:H2-K^b^-bearing Probe 2 caused a dramatic increase in ICAM-1 binding over time. Measurements from multiple RBC–T cell pairs reveals infrequent binding before the pMHC-bearing Probe 2 touched the T cell, followed by a gradual increase of *P*_*a*_ from 0.1 to 0.5 after a 100-s delay from the time of initial T cell–Probe 2 contact, and an eventual plateau of the LFA-1–ICAM-1 adhesion frequency, indicating global and sustained upregulation of LFA-1 binding affinity by TCR triggering (Fig. 2G, red). The ~6 µm distance between the stimulation location and the effector location indicates that the TCR signaling spreads globally throughout the cell surface. The divalent cation requirement for ligand binding and activation of LFA-1 was confirmed by replacing the L15 media in the reaction chamber with PBS without Ca^2+^ or Mg^2+^, which resulted in an adhesion curve indistinguishable from the nonspecific control curve obtained using Probe 1 coated with SA instead of ICAM-1 (Fig. 2G, blue and gray). It should be noted that different from the dual-ligand co-presentation case (Fig. 2A–C), the onset of LFA-1 upregulation by remote TCR signaling is on the scale of minutes instead of seconds, suggesting a more demanding threshold for such global (instead of local) inside-out signaling that must involve accumulation and dispersion of signaling intermediates over time and space. Note also that contacting T cells with Probe 2 coated with OVA:H2-K^b^α3A2 to prevent CD8 binding induced only a mild and transient increase in the frequency of T cell adhesion to the ICAM-1-bearing Probe 1 (Fig. 2G, purple). This again indicates a more stringent signaling requirement for global LFA-1 activation and is consistent with previous reports that CD8 plays an important role in TCR-induced lymphocyte adhesion strengthening^18^.

### Analyzing temporal crosstalk between GPIb and GPIIb-IIIa on a platelet

During the initial stage of hemostasis and thrombosis, mechanically modulated platelet adhesion occurs in two temporally separated stages in sequence: 1) Initial tethering stage, during which blood flow enhances attachment of platelets to and slows their translocation on the injured vascular surface through the interaction of platelet receptor GPIb to the A1 domain of the plasma protein von Willebrand factor (VWF)^33^; 2) Adhesion reinforcement stage, during which mechanosensing via A1–GPIb interaction triggers biochemical signals (i.e. Ca^2+^ flux) that activate the integrin receptor GPIIb-IIIa to further enhance platelet adhesion by binding to VWF^6,7^ and other plasma proteins such as fibronectin (FN)^34^. To dissect the temporal coordination between GPIb binding and GPIIb-IIIa upregulation, we wished to ask two specific questions: 1) whether the second receptor upregulation would sustain after removal of the stimulation of the first receptor; and 2) whether the GPIIb-IIIa affinity increase would correlate with the strength of the GPIb induced mechanosignals (i.e. Ca^2+^ levels).

**Figure 3:**
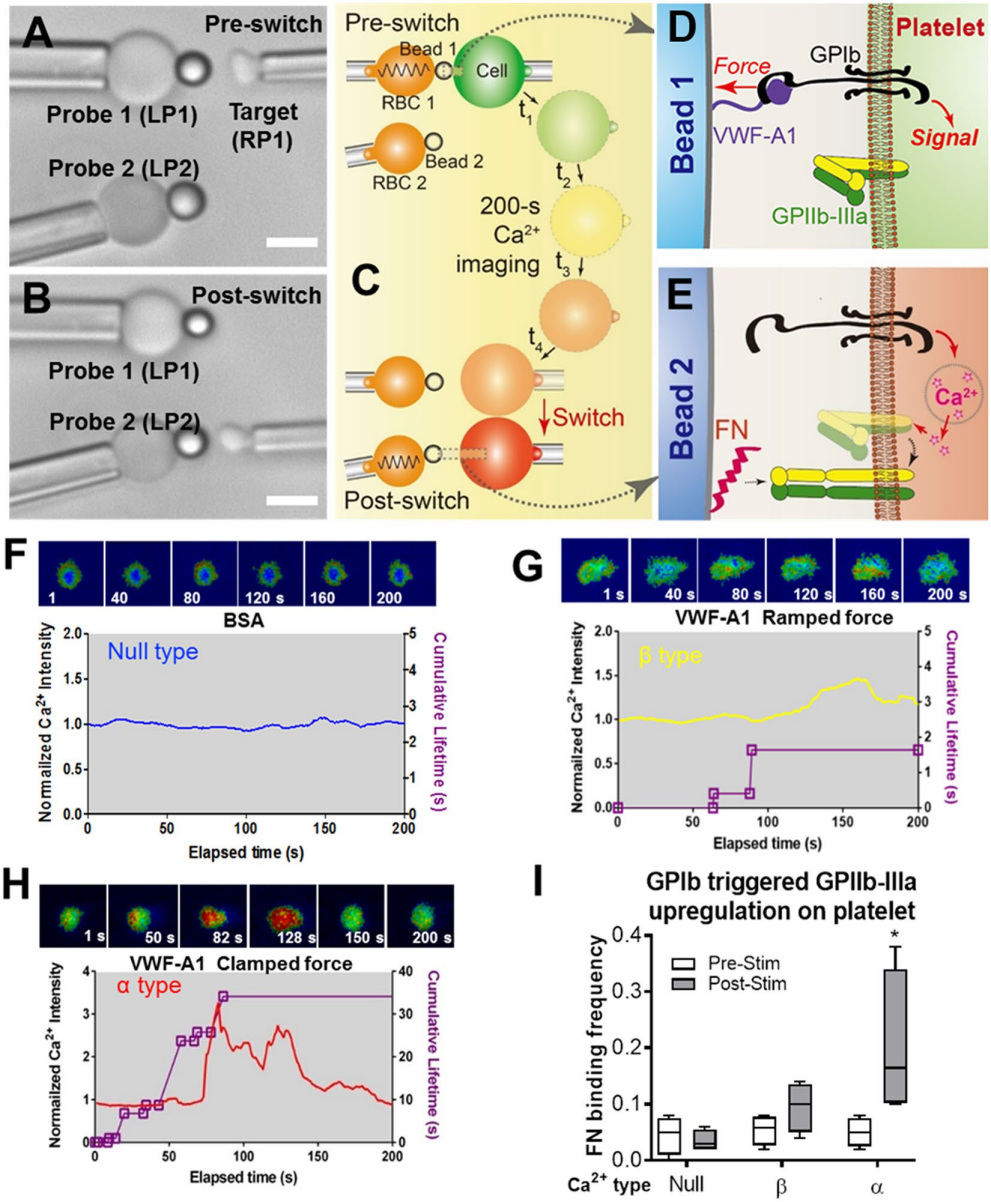
Analyzing temporal crosstalk between GPIb and GPIIb-IIIa. (**A,B**) dBFP setup for switching between interactions of the target cell with two immobilized ligands. Two beads functionalized with distinct proteins were attached to the apexes of two RBCs respectively aspirated by LP1 and LP2 to form Probes 1 and 2. A platelet held by RP1 was aligned against one probe at a time to sequentially engage two ligands, designated as the pre- (**A**) and post- (**B**) switch positions, respectively. Scale bars are 5 μm. (**C–E**) Schematics of the switching assay for studying temporal crosstalk within a platelet dual-receptor system (GPIb to GPIIb-IIIa). In the pre-switch position, the platelet was driven to repeatedly engage the first receptor GPIb with its ligand VWF-A1 coated on Probe 1 and exert a controlled pulling force on the A1–GPIb bonds (**C**, top; **D**). Intraplatelet Ca^2+^ signals triggered by GPIb mechanoreception were observed for 200 s (C, color gradient transition, middle). The subsequent upregulation of GPIIb-IIIa was probed by its ligand fibronectin (FN) on Probe 2 (C, bottom; E). (**F–H**) Concurrent measurement of platelet GPIb (receptor 1) triggered Ca^2+^ flux and bond kinetics. Top: Representative epi-fluorescence pseudo-colored images of intraplatelet Ca^2+^ in a platelet touched by BSA (non-adhesive control) (**F**) and VWF-A1 under ramped (1000 pN/s) (**G**) and clamped (25 pN) **(H)** forces at indicated times. Bottom: Representative time courses of normalized Ca^2+^ intensity (color matched curves and legends). The concurrent measurements of bond lifetime events (symbol) and the cumulative lifetime (purple curve) for the platelet exhibiting Ca^2+^ fluxes are overlaid. **I**. Pre- and Post-stimulation adhesion frequencies of platelet GPIIb-IIIa (receptor 2) binding to beads functionalized with FN. *n* ≥ 5 platelets were tested for each condition. *denotes *p* < 0.01 by Student t-test.

The dBFP system was used to address these questions. A human platelet, aspirated by RP1, was tested sequentially by two force probes aligned in parallel in the reaction chamber (Fig. 3A-C), formed by aspirating two RBCs by LP1 and LP2, respectively, and attaching one probe bead to the apex of each RBC using RP2 (Supplementary Fig. S2). The two beads were respectively coated with A1 and FN, the respective ligands for GPIb and GPIIb-IIIa (Fig. 3D,E). To observe intraplatelet Ca^2+^ signals triggered by GPIb mechanoreception, the platelets were preloaded with a fluorescent dye (Fura-2 AM) and the simultaneous Ca^2+^ ratiometric imaging optics were integrated as previously described^13,24,30^.

To separate engagements of the two receptors in time, we first drove the platelet to repeatedly touch Probe 1 coated with A1 (or BSA as a control) (Fig. 3A,D) with defined force waveforms programed within BFP touch cycles (Supplementary Fig. S3). For each platelet, the observed calcium trace was overlaid with the sequential binding events, bond lifetimes, and their accumulation (Fig. 3F–H). In the 200-s observation time window, three types of intraplatelet Ca^2+^ signals were observed as previously characterized^24^. These are: 1) null-type Ca^2+^, featured by a basal trace with a minimum intensity increase (Fig. 3F), which was observed on platelets touched by BSA coated beads with which no bond was formed; 2) β-type Ca^2+^, featured by signals fluctuating around the baseline or gradually increasing to an intermediate level (Fig. 3G), which was observed on platelets touched by beads bearing A1 with which a sequence of intermittent single brief bonds was induced by ramped forces (Supplementary Fig. S3A); 3) α-type Ca^2+^ with a much higher intensity, featured by an initial latent phase followed by a spike with a quick decay (Fig. 3H), which was observed on platelets that formed at least one durable bond with VWF-A1 under 25 pN clamped force (Supplementary Fig. S3B).

After the intraplatelet Ca^2+^ reached its peak and started to decay, we quickly (within 30 s) switched the platelet to contact Probe 2 coated with FN to measure the frequency of GPIIb-IIIa binding as a consequence of inside-out signaling that upregulated the integrin affinity (Fig. 3C). To correlate the integrin affinity changes with the signal types from GPIb mechanoreception, we compared the FN adhesion frequencies before and after the GPIb triggering and grouped them according to the type of calcium elicited. Interestingly, only those platelets that displayed α-type Ca^2+^ showed significantly upregulated integrin binding, whereas neither null- nor β-type did so (Fig. 3I). This is consistent with the previous observation that α-type Ca^2+^ serves as a platelet activating signal^24^ and indicates that GPIIb-IIIa upregulation is sustained for minute time elapse after GPIb triggered calcium spike has returned to the basal level. Our results are also consistent with the widely accepted model that GPIIb-IIIa assumes a low-affinity state on resting platelets^35^. During the arterial thrombosis with high shear conditions, the mechanoreception via GPIb upregulates the binding activity of GPIIb-IIIa to arrest platelets from translocation^6,7^. Thus, the dBFP enabled us to control the signal reception by GPIb, visualize the signal transduction inside the cell, and examine the signal effect on GPIIb-IIIa on a single platelet in one setup.

### Analyzing temporal requirement of signaling by ADP receptor on a platelet

**Figure 4:**
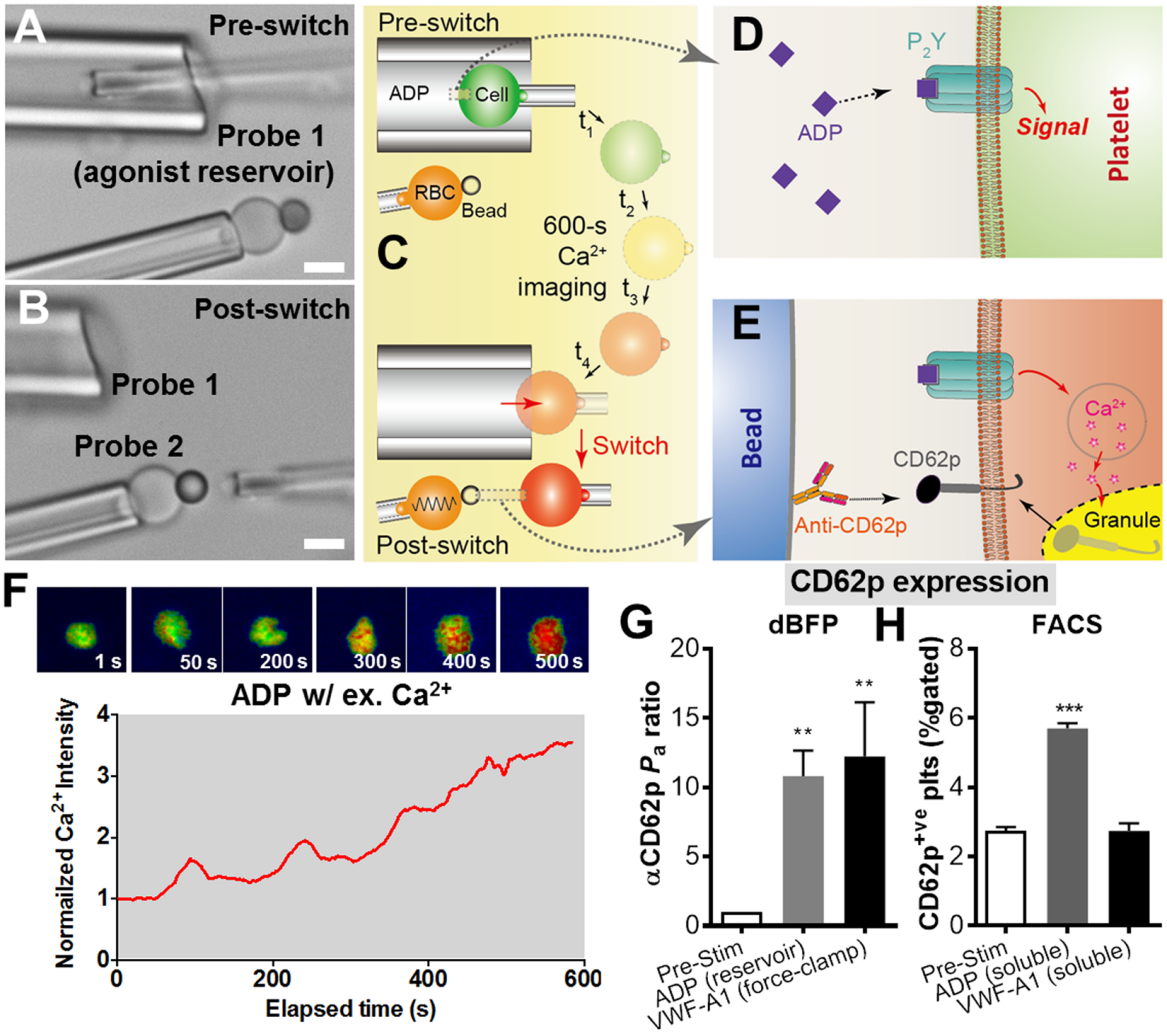
Analyzing temporal crosstalk between P_2_Y receptors and CD62p. (**A,B**) Setup for switching between interactions of the target cell with a soluble and an immobilized ligand. It is similar to that in Fig. 3A, B in both the pre- (**A**) and post- (**B**) switch positions except that Probe 1 is replaced by an open micropipette of large orifice (5–10 μm) as an agonist tank. Scale bars are 5 μm. (**C–E**) Schematics of the switch assay for investigating the upregulation of an activation marker (CD62p) by signaling via an agonist receptor (P_2_Y). An aspired platelet was immersed into the tank micropipette filled with ADP (50 µM) in the pre-switch position (C, top), which activates the platelet through P2Y receptors (**D**). The Ca^2+^ flux inside the stimulated platelet was monitored for 600 s (C, middle), followed by the switching to examine the platelet activation marker CD62p by an anti-CD62p antibody coated on Probe 2 (E). (**F**) Representative normalized Ca^2+^ level vs. time. (**G**) Adhesion frequency (*P*_a_) ratio between Post- vs Pre-stimulation for platelet CD62p binding to beads functionalized with the anti-CD62p antibody (AK4). *P*_a_ ratio for the “Pre-Stim” group equals 1. The results of stimulation by ADP tank immersion and immobilized VWF-A1 under a 25 pN clamped force (as set up in Fig. 3) are compared. (**H**) FACS measurements of platelet CD62p expression between Post- vs Pre-stimulation by soluble ADP (50 µM) and VWF-A1 (100 µg/ml) (see Methods). Results depict the % of gated platelets positive for anti-CD62p antibody binding. Data were presented as the mean ± s.e.m. from n ≥ 3 platelets for dBFP and n = 3 experiments for FACS under each condition. ** and *** denote *p* < 0.01 and 0.001, respectively, by Student t-test.

Crosstalk occurs between receptors that bind immobilized ligands at the two-dimensional cell-cell or cell-matrix junction, as in the previous two cases. In addition, many signaling events are initiated by soluble agonist binding to one cell surface receptor species, which upregulates the expression or function of another receptor species. At the later stage of hemostasis and thrombosis, for example, platelet plug formation requires potent stimulation by soluble agonists (e.g., ADP, thrombin, and/or TxA2), which leads to the expression of P-selectin (CD62p, released by α granule exocytosis) as a key marker of platelet activation^8,29^. To illustrate the utility of dBFP in analyzing dual receptor crosstalk of this kind, we replaced Probe 1 of the dual force probe setup with an open micropipette of large orifice (6–10 µm) and used it as an agonist tank by prefilling it with ADP (Fig. 4A). In the pre-switch position, the aspirated platelet was immersed into Probe 1 and incubated for 10 min to allow ADP to bind the P_2_Y receptors on platelet for long enough time (Fig. 4A,C,D). During the subsequent 600-s observation time window, high and sustained intraplatelet Ca^2+^ signals were observed (Fig. 4F), indicating that the platelet was being activated^29^.

To read out the consequence of ADP stimulation in terms of upregulation of another receptor, we analyzed the CD62p expression by moving the aspirated platelet out of the Probe 1 tank and then reorienting it against Probe 2 (Fig. 4B,C) functionalized with an anti-CD62p antibody (αCD62p, Fig. 4E). Adhesion frequency assay was run to measure binding, which reported the CD62p expression level. Comparing to the pre-stimulated platelets, the post-stimulated platelets displayed >10-fold higher binding to the αCD62p bearing Probe 2 (Fig. 4G), which is consistent with the effect of ADP in activating platelets.

### Comparing temporal crosstalks initiated by ADP and VWF-A1 on a platelet

To compare with consequences of receptor signaling initiated by binding of soluble vs. immobilized ligands, we also measured CD62p expression following signaling via GPIb triggered by engaging VWF-A1 under a clamped force. This was done using the same setup and procedures as in the study of temporal crosstalk between GPIb and GPIIb-IIIa (Fig. 3). The only difference was to coat Probe 2 with αCD62p instead of FN. Interestingly, the anti-CD62p antibody binding was also enhanced by >10 times (Fig. 4G).

To demonstrate the utility of our dBFP in studying juxtacrine signaling and receptor-mediated mechanosensing, we used flow cytometry (FACS) experiments to compare the CD62p expressions respectively induced by binding of ADP and soluble VWF-A1 to platelets. In contrast to the dBFP experiments, soluble ADP significantly shifted CD62p positive platelet populations from 2.5% to 6% whereas soluble VWF-A1 showed no effect at all (Fig. 4H). It should be noted that the soluble VWF-A1 used is able to bind GPIb on platelets^36^. Our results agree with the recent report that GPIb-induced platelet surface expression of CD62p requires force^28^. The low percentage shifting (<4%) of CD62p positive platelets in FACS reflects the widely accepted property of ADP as a weak agonist^29^. The much higher increase (>10 times) of αCD62p binding is due to the dBFP’s mechanical readout, which is capable of detecting a single CD62p bond. By comparison, a small increase in CD62p expression was shown as only a small shift in the fluorescence histogram in the FACS assay. This example illustrates the advantage of the dBFP over conventional approaches in its ultrasensitivity to examine both biochemical (P_2_Y/CD62p) and biomechanical (GPIb/CD62p) signal crosstalks.

## Discussion

This paper described the dBFP as a versatile experimental platform for mechanical analysis of signal crosstalk between multiple molecular species on a living cell. Three single applications that use the dBFP instrument were illustrated using four dual receptor biological systems with biological interests in two different fields: immunology and hemostasis/thrombosis. Although the general conclusions of our experiments are known in qualitative terms from previous studies using conventional methods, more quantitative details have been obtained by the present work using the dBFP.

The dBFP technique enables the investigation of the cooperative binding of multiple receptor–ligand species located in the proximal or separate regions of the same cell surface using mixture presentation or separate presentation of multiple ligands, respectively. In the case of LFA-1 inside-out signaling via TCR triggering, the dBFP provided the spatiotemporal characteristics of this crosstalk that were unable to be resolved using conventional static binding assay or fluorescent imaging techniques^4,5^. We observed an upregulation of LFA-1–ICAM-1 binding triggered by TCR–pMHC binding, which required ~100 s to occur and spanned ~6 µm distance across the cell membrane. By comparison, using mixture ligand coating the cooperative binding between the two receptor–ligand species took place almost instantaneously (with only ~2 s delay). The distinct kinetics of the two crosstalk cases of the same dual receptor system but over different distances is revealing. The much faster upregulation of LFA-1 binding by TCR signaling in the case of co-presenting pMHC and ICAM-1 is intuitive because mixed ligands would allow engagement of two receptor species residing in close proximity within a structural platform, e.g., membrane domain or signalosome, to facilitate signal crosstalk. Separate presentation of pMHC and ICAM-1 prevents signal transduction within such putative structural platforms, thereby greatly diminishing its efficiency. Compared to most fluorescent studies of TCR and LFA-1 in the immunological synapse, the two dBFP setups were able to isolate the net effects of LFA-1 upregulation by TCR, and also reveal a global modulation that was not confined by the immunological synapse. The setups and analytical framework could be applied to other dual receptor–ligand systems to expand our understanding of the molecular crosstalk in synapses and kinapses^3^.

In addition, the observation by dBFP of a delay time for LFA-1 activation (~100 s) demonstrates its ability to measure the required time for signaling events to occur after the stimulation. This is of special interest to the study of complex signaling pathways that involve multiple steps. By defining the temporal sequence of different signal readouts, including fluxes of intracellular molecules and up/down-regulation of surface receptors, one can determine the upstream/downstream relationship of signaling events. The limitation of the ‘temporal crosstalk’ setup lies in the time required for the experimentalist to perform the ‘switch’ maneuver, which is no less than 30 seconds. Signaling events taken less than 30 seconds would be hard to capture.

Previous population studies using cone-and-plate viscometers^37^, bulk flow chambers^6,7,38^ and microfludics devices^39^ have documented the mechanical signal crosstalk between GPIb and GPIIb-IIIa. However, the control over the incoming signal is very limited in these studies. Relative contributions from each signaling component is difficult to dissect, and none of them could perform single-cell manipulation. No decisive conclusion can be drawn for the mechanisms of signal transduction at molecular scale. By comparison, the dBFP is capable of precisely controlling and varying the timing when a cell begins and ends its contact with a ligand-bearing surface or with soluble agonists, the speed of the cell approaching to and detaching from the ligand-bearing surface, the duration and area of contact, the intermission period between consecutive contacts, and the waveform of force exerted on a single receptor by an engaged ligand.

A large population of cells in suspension is usually needed in the conventional approaches for studying signal transduction, regardless of whether these are biochemical (e.g., western blot and flow cytometry) or biomechanical (e.g., flow chamber and viscometers) based. This inevitably allows cells to communicate with each other by releasing soluble agonists upon stimulation. For example, adjacent platelets have been observed to undergo “intercellular calcium communication”^40^, providing a positive feedback mechanism that prompts global activation of platelets. In population studies, therefore, what is observed in a cell’s response to external stimulation is always “contaminated” by collateral responses of paracrine signaling. However, in the dBFP assay this problem is prevented by stimulating a single cell at a time: in the agonist stimulation, a single cell was immersed in the agonist-filled micropipette; whereas in the mechanical stimulation, receptor–ligand bonds were also formed on one cell only. Platelets were observed to induce fast Ca^2+^ onset upon addition of ADP in previous population studies^41,42^, different from the slower Ca^2+^ onset observed in this work (Fig. 4F). This could be due to the lack of intercellular communications in the isolated stimulation by nature of our dBFP experiments, which slowed down the platelet activation process. This suggests another advantage of the dBFP: it can stimulate a single cell with soluble agonists without the influence of paracrine signaling from neighboring cells.

Single-molecule imaging is a fluorescently based method for detecting receptor–ligand binding events across a hybrid junction between a cell and a supported lipid bilayer^14^. This method can track interaction of one receptor–ligand species at a time, or concurrent interactions of multiple receptor–ligand species all at once, providing a complementary tool to the dBFP for studying signal crosstalk between multiple receptors. Compared with our dBFP method, however, it has several disadvantages: 1) The cell and the surface will stay in contact for all time, meaning that the contact time cannot be used as a variable to manipulate the amount of stimulation the cell receives. Instead, the density of ligand is titrated to manipulate the amount of stimulation. 2) Bonding forces cannot be controlled and externally applied. 3) The ligand has to be fluorescently labeled and inserted into a lipid bilayer. Therefore the same setup cannot be applied to agonist receptors.

Scientists have long been using genetic manipulation as a powerful tool to study biological systems, e.g., making gain- or loss-of-function mutations to perturb the molecules of a biological process to gain insights into the underlying mechanism (i.e. the players of the game). Our study shows that mechanical manipulation can also be used as a powerful tool to perturb the physical variables of the biological process (i.e. the rules of the game). The dBFP is an ultrasensitive force technique for mechanical manipulation. As such it can help reveal molecular mechanisms underlying the signal crosstalk between two or more types of receptors, particularly when space and time are limiting variables in the signal transduction process. Potential applications of the dBFP may include probing other dual receptor systems such as selectins/β_2_ integrins in leukocyte adhesion^43^ and TCR/CD8 or TCR/CD4 in T cell activation^18^. By replacing the RBC on Probe 2 with a different cell type, the dBFP can be used to probe cooperativity in cell-cell interaction following initial signal activation, such as the cases of platelet–leukocyte interaction^44^ and T-cell–antigen presenting cell interaction^45^. Furthermore, one can imagine that when more micropipettes or other holding mechanisms (e.g. optical traps) are installed in the system, more dimensions of stimulation (either mechanical or chemical) can be applied and readouts can be obtained sequentially and/or concurrently provided with enough space. This will be a powerful tool to understand more complicated crosstalk problems involving more than two receptor species and to elucidate their synergistic, competitive and/or suppressive interrelationship.

## Method

### Protein and reagents

Recombinant mouse ICAM-1 was from R&D Systems and biotinylated with a Pierce EZ-Link^®^ Biotinylation Kits (ThermoFisher Scientific) by following the manufacturer’s instruction. Biotinylated recombinant pMHC monomers OVA:H2-K^b^ and OVA:H2-K^b^α3A2 were from the National Institutes of Health Tetramer Core Facility at Emory University. Recombinant VWF-A1 monomeric domain (residues 1238–1471) was a kind gift from Miguel Cruz (Baylor College of Medicine, Houston, TX) as described previously^24,39,46^. Human fibronectin (cat # 33016015; ThermoFisher Scientific), anti-CD62p-FITC antibody (AK4; BD Bioscience), calcium indicator Fura2-AM (Life Technologies) were purchased. All other reagents were purchased from Sigma-Aldrich unless stated otherwise.

### Functionalization of glass beads

Ligands or antibodies were coated on glass beads as previously described^13,30,47^. Briefly, proteins were first covalently modified with maleimide-PEG3500-NHS (molecular weight ~3,500 Da; JenKem, TX) in carbonate/bicarbonate buffer (pH 8.5). Borosilicate beads (2-µm in diameter, cat # 9002; ThermoFisher Scientific) were silanized and covalently coupled with 3-Mercaptopropyltrimethoxysilane (Uct Specialties, llc, PA). The maleimide-modified proteins together with streptavidin-maleimide were covalently linked to the glass beads in phosphate buffer (pH 6.8). After overnight incubation and resuspending in phosphate buffer with 1% HSA, beads were ready for immediate use in BFP experiments.

### Red blood cell and platelet isolation and preparation

Human RBCs and platelets for all experiments were isolated from venous whole blood of healthy volunteers by a certified venesectionist according to protocols approved by the Institutional Review Board of Georgia Institute of Technology as previously described^13,30^. All human donor blood samples were obtained with informed consent and all experiments were performed in accordance with relevant guidelines and regulations. To make adhesion assay probe for dRMP experiment (Fig. 2), RBCs were washed and biotinylated with Biotin-X-NHS^30,48^. To make force probe in dBFP assay (Figs 3 and 4), RBCs were washed, biotinylated with Biotin-PEG3500-NHS (JenKem, TX), swollen with nystatin and stored (up to 2 weeks) for experiments^13,30^.

To purify platelets, 2.57 ml whole blood was added to 0.43 ml ACD buffer (6.25 g sodium citrate, 3.1 g citric acid anhidrous, 3.4 g D-glucose in 250 ml H_2_O, pH 6.7). The ACD blood was then centrifuged at 150 g for 15 min without brake at room temperature. The platelet rich plasma was extracted and centrifuged again at 900 g for another 10 min. In selected Ca^2+^ imaging experiments, 0.5 ml platelet rich plasma was extracted and incubated with Fura-2-AM at 30 μM for 30 min before the second centrifugation. The platelet pellet was resuspended in the Hepes-Tyrode buffer (134 mM NaCl, 12 mM NaHCO3, 2.9 mM KCl, 0.34 mM sodium phosphate monobasic, 5 mM HEPES, and 5 mM glucose, 1% BSA, pH 7.4)^24,39,49^.

### T cell purification

OT1 transgenic mice were housed at the Georgia Institute of Technology Department of Animal Resources facility followed by a protocol approved by Institutional Animal Care and Use Committee (IACUC). All mouse samples were obtained with informed consent and all experiments were performed in accordance with relevant guidelines and regulations. Naïve CD8^+^ T cells were purified from 6–8 week-old mouse spleens using a CD8^+^ negative purification kit (Stem Cell Technology) as previously described^30^. Cells were stored on ice in R10 media consisting RPMI 1640 (Cellgro) supplemented with 10% FBS (Cellgro), 100 U/ml penicillin, 100 µg/ml streptomycin, 2 mM L-glutamine, 20 mM HEPES.

### dBFP instrumentation

The dBFP was an upgrade from the previously reported fluorescence BFP, which was built on a Nikon inverted microscope (TiE) with a 40X/0.75 objective (Supplementary Fig. S1)^13^. Briefly, an integrated light path was used for simultaneous ratiometric calcium imaging (i.e. Fura-2 dual excitation configuration) and tracking force transducer position from the bright-field view (Fig. 3). The major improvement was made around the experiment stage that consists of three components from the left to the right: 1) The left micropipette sets LP1 and LP2 are anchored to a 3D micrometer precision translational stage (462-XYZ-M, Newport) through a double-pipette holder set (HD-21, Narishgie) (Fig. 1C). 2) The other two micropipettes are placed on the right. RP1 is mounted by a 3D piezoelectric translator (PZT) (M-105.3 P and P-753.1CD, Physik Instrumente) to be driven by a computer program for nanometer-precision movement. RP2 is connected to a hydraulic micromanipulator (PH400, Karl Suss) and positioned against RP1 with 45-degree angle (Fig. 1E). 3) A reaction chamber in the middle filled with experimental buffer into which the micropipettes were inserted and beads and cells were injected (Fig. 1D). All four micropipettes are connected with their corresponding homemade manometers (Supplementary Fig. S1A). By adjusting the reservoir height of a manometer, one can control the inner pressure of the micropipettes to aspirate or blow away a bead or a cell during experiments.

### dBFP assembly

The two micropipettes LP1 and LP2 were used to aspirate two swollen biotinylated human RBCs (Supplementary Fig. S2A). To form two biomembrane force probes, Bead 1 and 2, coated with respective ligands and antibodies of interest as well as streptavidin, were sequentially aspirated by RP2 and placed onto the apexes of RBC 1 and 2 (Supplementary Fig. S2A,B). A target cell (e.g. platelet) that displays the signaling machinery of interest (Fig. 3D,E) was aspirated by RP1 and aligned against Bead 1 (Supplementary Fig. S2C). In an experiment, RP1 was driven by the PZT to move precisely as programmed, touching in and out of contact with the force probes. The 3-way PZT allows maneuver of the target cell’s position in all three dimensions by manually adjusting the PZT controller. Therefore, this configuration allows us to switch the interactions of the same cell between two ligands presented on two separate force probes (Fig. 3).

Similar to the BFP, the bond force in the dual force probe assembly is determined by multiplying the RBC deformation and the spring constant (i.e. 0.3 pN/nm in this study) as previously described^13,30,50^. The RBC deformation can be regarded as the axial movement of the probe bead tracked by a high-speed camera^13^. Adhesion frequencies from these cycles were kept low (<20%) by adjusting the ligand density as required for the platelet to be pulled predominantly (>89%) by a single bond^31^.

### Force-clamp and force-ramp assays

These assays have been previously described^1,13,30^. In these assays, the RP1 aspirated cell is driven to repeatedly contact the probe bead for 2 s and retract at a constant speed (3.33 μm/s). Multiplying the BFP spring constant (0.3 pN/nm), this translates to a linearly increasing force at a constant loading rate (1,000 pN/s). For the force-ramp assay (Supplementary Fig. S3A), the system was programmed to allow continuous cell retraction until bond rupture without holding at a constant force. For the force-clamp assay (Supplementary Fig. S3B), upon detection of a bond event, the cell would be retracted to a pre-determined position and paused to exert a desired clamped force (e.g. 25 pN) on the bond to wait for bond dissociation. After that the RP1 returns to the original position to complete the cycle. Each cell was interrogated for a continuous time of 200 s to generate a force trace exemplified in Supplementary Fig. S3 right column before replacement for a new set of cell and probe. Lifetimes were measured from the instant when the force reached the desired level to the instant of bond dissociation (Supplementary Fig. S3B *right column*)^24,38^.

### Agonist stimulation in the tank micropipette

In experiments that require pre-exposing the aspirated cell to a soluble reactant, the LP1 was fabricated as an open micropipette of a large orifice (6–10 µm) and prefilled with an soluble agonist ADP using MicroFil (MF34G-5; World Precision Instruments, Sarasota, FL) (Fig. 4A). To minimize diffusion or even minuscule convection that could lead to cross-contamination with the medium surrounding this pipette, apyrase, an ADP scavenger, was added into the chamber buffer to eliminate the ADP that leaks from the large micropipette to the chamber. To maintain the ADP concentration in the tank, we inserted the platelet deep inside the large micropipette, so that it would not be easily influenced by the medium diffusion from the large micropipette mount. We also kept a very low positive pressure in the large micropipette to slowly eject the ADP solution out to continuously get rid of the solution at the tip of the micropipette, where ADP was diluted by the chamber buffer and chelated by apyrase. By doing so, the platelet inserted into the tank micropipette would be immersed in the correct concentration of ADP during the incubation time.

### Flow cytometry (FACS)

For CD62p expression analysis, isolated human platelets in Tyrode’s buffer (5 × 10^7^ /ml) were incubated with anti-CD62p-FITC monoclonal antibody (AK4, BD Biosciences) according to the manufacturer’s instructions. For stimulating conditions, the platelets were mixed with either soluble ADP (50 µM) or VWF-A1 (100 µg/ml) for 10 min at 37 °C, then diluted with Tyrode’s buffer and analysed on a FACSCalibur® flow cytometer (BD Biosciences). The percentage of gated platelets positive for antibody binding was measured as the expression level. The gate was set at the 2% of the histogram for platelets mixed with IgM-FITC κ isotype control (314506; BioLegend).

## Electronic supplementary material

Supplementary Figures
