# Supplementary material for: Dual Biomembrane Force Probe enables single-cell mechanical analysis of signal crosstalk between multiple molecular species
Source: Sci Rep. 2017 Oct 27;7:14185. doi: 10.1038/s41598-017-13793-3 (PMC5660210; doi:10.1038/s41598-017-13793-3)
Supplement: Supplementary file 1 — Supplementary Figures [file 41598_2017_13793_MOESM1_ESM.pdf]

Supplementary Information

Dual Biomembrane Force Probe enables single-cell mechanical analysis of signal crosstalk between multiple molecular species

Lining Ju, Yunfeng Chen, Kaitao Li, Zhou Yuan, Baoyu Liu, Shaun P. Jackson and Cheng Zhu

Supplementary Figures

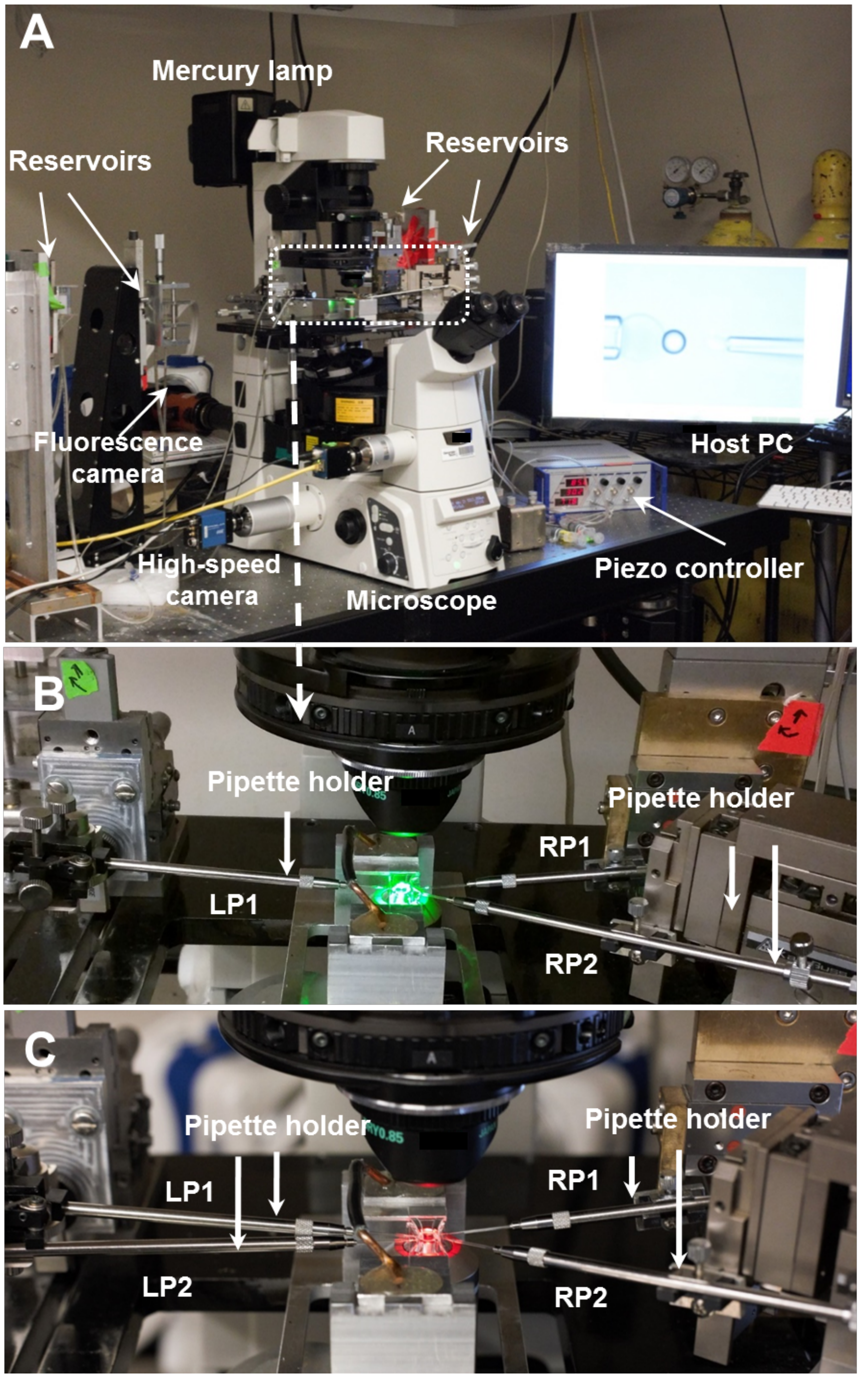

Supplementary Figure S1. Dual BFP instrumentations. A. An overview picture of the dBFP instrumentation setup. Core parts including the microscope, piezo controller and cameras were marked. **B, C.** Zoom-in configurations of spatial separation (B) and temporal separation (C) setups. Each micropipette and its holder are indicated.

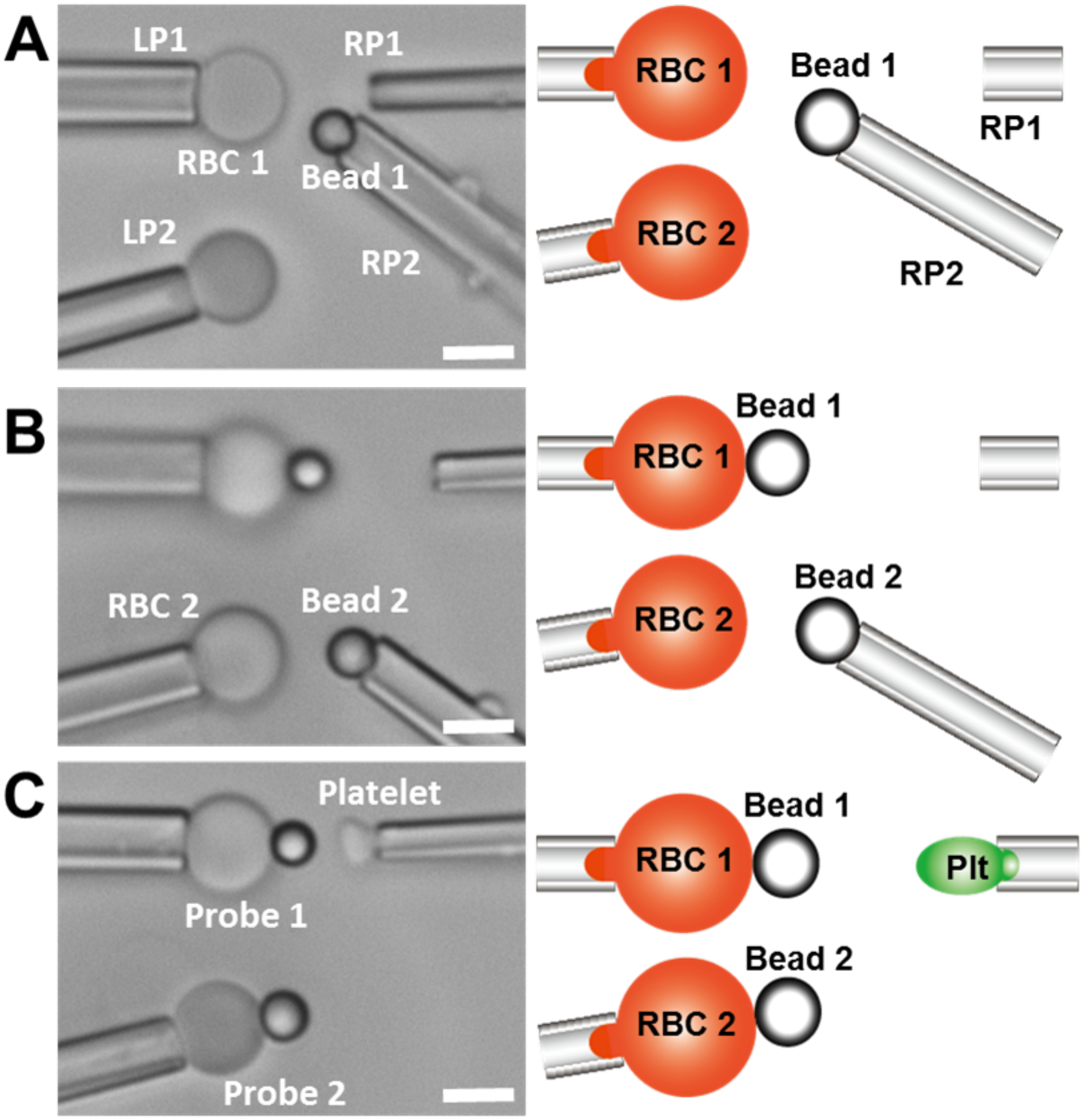

Supplementary Figure S2. Temporal dBFP operation procedure in micrographs and illustrative sketches. Micropipettes LP1 and LP2 each aspirated a RBC (RBCs 1 and 2). Micropipette RP2, as a helper, aspirated then delivered Bead 1 onto the apex of RBC 1 to assemble Probe 1. **B.** Similar steps were performed using RP2 to assemble Probe 2. **C.** Micropipette RP2 was moved out of the way (and view) while RP1 aspirated a platelet and aligned against Probe 1 to get an experiment ready. Scale bars = 5 μm in all micrographs.

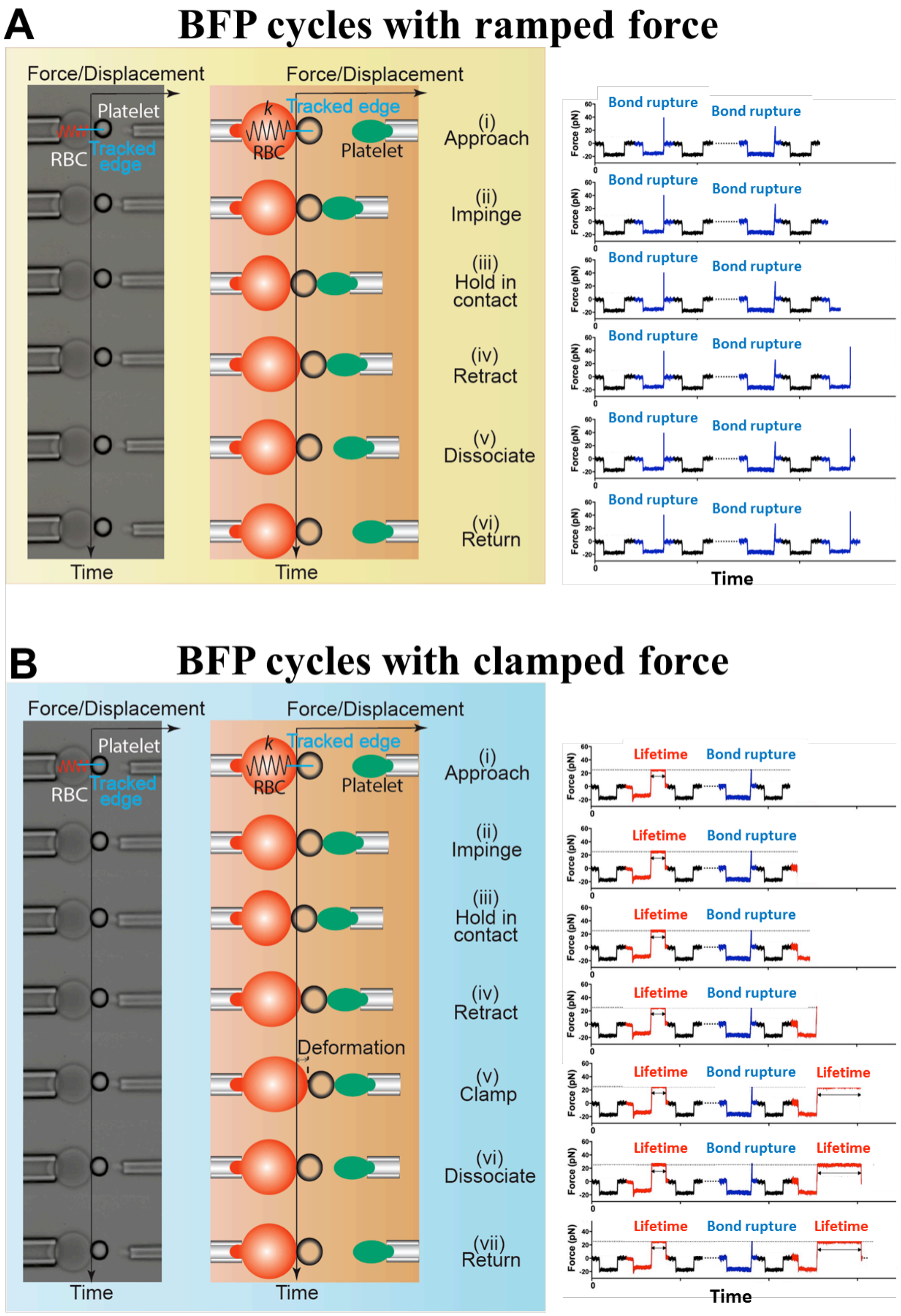

Supplementary Figure S3. BFP force-ramp and force-clamp assay procedures. Micrographs (left column), schematics (middle column) and force vs. time traces (right column) of one BFP test cycle with a ramped force (A, Step (i)-(vi)) or a clamped force (B, Step (i)-(vii)). Position (horizontal axis) of the probe bead attached to the apex of the RBC aspirated by the fixed micropipette (left columns) goes through the indicated steps as time (vertical axis) increases in response to the interaction with the target cell (i.e. platelet)

aspirated by the moving micropipette (right column) controlled by a computer. The origin of the horizontal axis indicates the resting position of the RBC apex when no force is applied to it. Upon contact the platelet interacts with the probe bead and changes its position (<0 by pushing and >0 by pulling), causing RBC deflection that can be modeled by a spring with a spring constant k to allow conversion of the deformation (Δd) to force ($= k \times \Delta d$). The probe bead position is tracked at a high temporal resolution (1,500 Hz) using a high-speed camera. The line edge tracker of the RBC apex position is shown in blue. Cycles produced different results are color-coded (black: no bond; blue: bond-rupture; red: bond-lifetime). For (B), 25pN clamped force was used.
